# Correction: Pérez-Prieto et al. Are Hamstring Grafts a Predisposing Factor to Infection in R-ACL Surgery? A Comparative In Vitro Study. *Pathogens* 2023, *12*, 761

**DOI:** 10.3390/pathogens15040447

**Published:** 2026-04-21

**Authors:** Daniel Pérez-Prieto, Svetlana Karbysheva, Andrej Trampuz, Oscar Fariñas, Joan C. Monllau, Ferran Corcoll

**Affiliations:** 1 Department of Traumatology and Orthopaedic Surgery, Hospital del Mar—Universitat Autònoma de Barcelona (UAB), 08003 Barcelona, Spain; dr.danielperezprieto@gmail.com (D.P.-P.); jmonllau@psmar.cat (J.C.M.); 2 Center for Musculoskeletal Surgery, Charite’—Universitätsmedizin Berlin, Corporate Member of Freie Universität Berlin, Humboldt-Universität zu Berlin and Berlin Institute of Health, 10117 Berlin, Germany; svetlana.karbysheva@charite.de (S.K.); andrej.trampuz@charite.de (A.T.); 3 Banc de Sang i Teixits de Catalunya, Barcelona (BST), 08005 Barcelona, Spain; ofarinas@bst.cat; 4 Departament de Ciències Morfològiques, Edifici M Facultat de Medicina Avinguda Can Domènech S/N Campus de la Universitat Autònoma de Barcelona (UAB) Bellaterra (Cerdanyola del Vallès), 08193 Barcelona, Spain

## Change in Author Order

In the original publication [1], the published author order was: “Ferran Corcoll ^1,^*, Daniel Pérez-Prieto ^1^, Svetlana Karbysheva ^2^, Andrej Trampuz ^2^, Oscar Fariñas ^3^ and Juan Carles Monllau ^1,4^”. 

The **correct author order** that should have been published is: 

“Daniel Pérez-Prieto ^1^, Svetlana Karbysheva ^2^, Andrej Trampuz ^2^, Oscar Fariñas ^3^, Joan C. Monllau ^1,4^ and Ferran Corcoll ^1,^*”. This error was overlooked by the authors during the proofreading stage. 

The corrected author contributions are unaffected. This correction was approved by the Academic Editor. The original publication has also been updated.
